# The cardiovascular safety of the empirical measurement of the seizure threshold in electroconvulsive therapy

**DOI:** 10.1192/pb.bp.112.038695

**Published:** 2015-02

**Authors:** Lindsay Mizen, Charles Morton, Allan Scott

**Affiliations:** 1 Royal Edinburgh Hospital; 2 Royal Infirmary of Edinburgh

## Abstract

**Aims and method** The Royal College of Psychiatrists’ Committee on Electroconvulsive Therapy (ECT) and Related Treatments advises the measurement of initial seizure threshold in all patients undergoing ECT if possible. The subconvulsive electrical stimulation inherent in this process is thought to increase the risk of bradycardia and therefore asystole. Our aim was to establish the prevalence of asystole (no heart beat for 5 or more seconds) during empirical measurement of seizure threshold in patients who had not received anticholinergic drugs, as we were unable to find any published reports of bradycardia or asystole prevalence under these conditions. The electrocardiogram traces of 50 such consecutive patients were analysed later.

**Results** Asystole occurred in 5% of stimulations. Each episode of asystole resolved spontaneously with no adverse outcomes. Contrary to expectations, asystole was no more prevalent in subconvulsive stimulations than in convulsive stimulations.

**Clinical implications** There was no evidence that the empirical measurement of the seizure threshold added to the cardiovascular risk of ECT.

The electrical stimulus of electroconvulsive therapy (ECT) may have effects other than the induction of cerebral seizure activity. It was recognised soon after the introduction of ECT that the stimulus could affect the heart, usually causing a bradycardia resulting from the electrical stimulation of the motor nucleus of the vagus nerve and nucleus ambiguus, within the medulla oblongata.^1^ This bradycardia can be severe and as long ago as 1978 the American Psychiatric Association (APA) stated that the bradycardia might even be prolonged enough to cause cardiac arrest.^2^ Although no references were given to support this concern, asystole and cardiac arrest have been reported since.^3^ The APA recommended the routine intravenous administration of an anticholinergic drug to reduce the risk of severe bradycardia, but this never became routine anaesthetic practice in the UK. The Royal College of Psychiatrists recognises that ‘severe bradycardia can usually but not invariably be prevented by pre-treatment with anticholinergic agents’ and that such vagolytic drugs are sometimes used to attenuate bradycardia, but it does not specifically recommend the routine administration of an anticholinergic drug.^4^

Evidence from case reports has been taken to mean that severe bradycardia is more likely after subconvulsive electrical stimulation; that is, stimulation that does not lead to generalised cerebral seizure activity.^1,2,4^ This is because seizure activity has an opposite effect on the heart, leading to tachycardia, whereas in subconvulsive stimulation the vagal effect is unopposed.^1^ Subconvulsive stimulation was uncommon in traditional ECT practice because fixed, high doses of electrical charge were delivered. Contemporary ECT machines can deliver adjustable doses and it is now recommended that the dose is titrated for the individual patient, particularly for bilateral ECT.^4^ The convulsive dose depends on the seizure threshold for the individual patient. This is measured empirically by stimulation with repeated, increasing doses of electrical charge at the start of the course of ECT. In contemporary practice, the vast majority of patients receive at least one subconvulsive stimulation at their first treatment in a course of ECT. The APA now notes that not all anaesthetists routinely use anticholinergic drugs, but also states that the bradycardia after subconvulsive stimulation is of ‘graver’ concern, and that many anaesthetists routinely administer such agents at treatments in which the measurement of seizure threshold is planned.^5^ Some ECT practitioners simply do not undertake empirical measurement of the seizure threshold in patients with a known history of cardiac disease.^6^

We aimed to investigate the prevalence of asystole during empirical measurement of seizure threshold in the absence of anticholinergic drugs.

## Method

We analysed the printed paper record of the electrocardiogram (ECG) taken during the first treatment of 53 consecutive patients referred for a new course of ECT at the Royal Edinburgh Hospital between 12 February 2008 and 5 March 2010. An individual patient was included only once. Three of these patients are not included in the present report because the anaesthetist administered an intravenous anticholinergic drug before ECT; in all cases this was because of previous excessive salivation during ECT. The present sample therefore consists of 50 from 53 consecutive referrals for ECT. The intravenous induction agent was etomidate in 38 patients and propofol in 12 patients. Muscle relaxation was always achieved by intravenous suxamethonium, after precurarisation with 5 mg atracurium. The vast majority of patients (*n* = 47) were treated with bilateral ECT; the remaining 3 received right unilateral ECT.

**Fig. 1:**
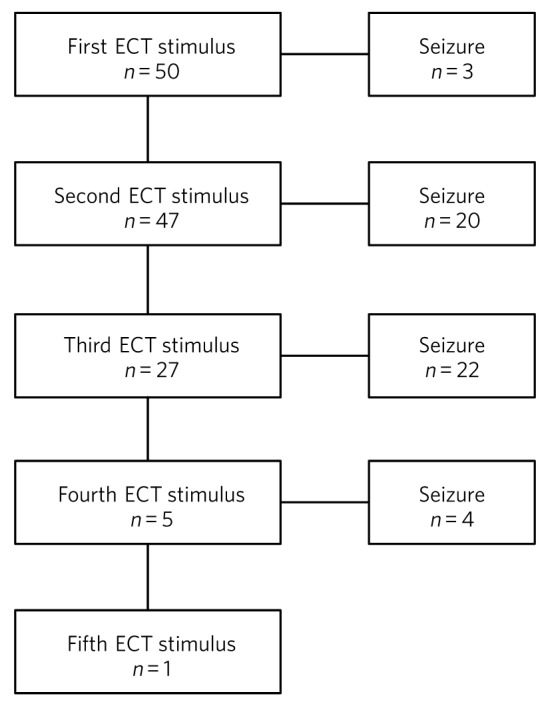
Numbers of patients in the sample receiving one or more ECT stimulations.

All treatments were administered using a Spectrum 5000M ECT machine, which has an inbuilt two-channel electroencephalogram (EEG) facility to measure cerebral seizure activity. The protocol for the empirical measurement of the seizure threshold at the first treatment was as follows: the first stimulation was with 4% (46 mC), and if cerebral seizure activity did not ensue, then a second stimulation was given with 7% (81 mC). In patients prescribed an anti-epileptic drug, the first stimulation was with 9% (104 mC) and if no cerebral seizure activity ensued, then a second stimulation was given with 18% (207 mC). For both groups of patients the protocol included doses for third and subsequent stimulations, when necessary. The flow chart presented in Fig. 1 shows the number of patients receiving one or more stimulations.

Our clinic participates in the Scottish national audit of ECT practice, coordinated and reported by NHS National Services Scotland. This paper includes demographic, episodic and treatment data routinely recorded and reported. The only change made to our routine data collection was to print a paper record of the ECG. These paper records were given a coded number by the ECT consultant (A.S.) and then stored outside the ECT clinic until they were read by an independent rater (L.M.); clinical and treatment information was masked to the rater. We used ECG lead II and the paper record ran at 25 mm/s.

**Fig. 2:**
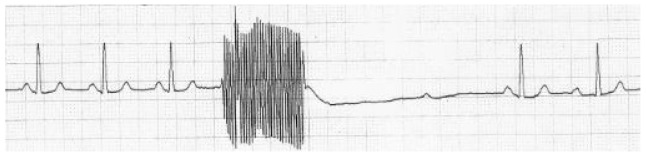
ECG with electrical stimulus artefact followed by a period of asystole.

The point when bradycardia becomes asystole is arbitrary. The criterion that 5 s (125 mm on the paper record) without a QRS complex constitutes asystole is taken from a previous report.^7^ Its authors also conducted a secondary analysis of patients in whom the asystole lasted as long as 7 s and so we also considered a secondary analysis with this definition of asystole. The analysis of the paper records was standardised by discussion among the present authors before analysis. The electrical stimulation led to a characteristic type of artefact on the paper record (Fig. 2) and the time from the end of this artefact (that is, the end of electrical stimulation) to the time of the first QRS complex was measured. The morphology of this complex had to be the same morphology as complexes before electrical stimulation, which meant ectopic beats were disregarded. If the independent rater was uncertain about the measurement of this time, she consulted C.M. and the time was agreed between these raters; patient details remained masked to L.M., and C.M. was shown only the paper record and any other clinical or treatment information was masked. The results were analysed using Microsoft Office Excel 2007 and StatsDirect Version 2.7.9 for Windows. As the inter-subject variance (between different patients) was

likely to be higher than the intra-subject variance (multiple stimulations in one patient), each electrical stimulation was considered as an independent measure. Convulsive and subconvulsive stimulations were compared using Fisher’s exact test and the ‘exact’ or ‘Clobber-Pearson’ method was used to calculate 95% confidence intervals of proportions.^8^ The episodes of asystole in different stimulations were compared using paired *t*-tests.

It is the policy of NHS Scotland to withhold from publication data that may identify individual patients.^9^ This report respects this principle.

## Results

As shown in Table 1, the sample consisted of 37 women and 13 men (mean age 57.4, range 22–87 years). The most prevalent primary psychiatric diagnosis was severe depression with (*n* = 18), or without (*n* = 16), psychosis; 6 patients had moderate depression and 7 were diagnosed with other types of depressive disorder. Six of the total group of patients with depression had bipolar affective disorder. Schizophrenia was the primary diagnosis in 2 patients.

The majority of patients (*n* = 38) were prescribed a combination of psychotropic drugs, including tricyclic antidepressants (*n* = 6) and lithium carbonate (*n* = 7); 8 patients were prescribed monotherapy, 6 with an antidepressant drug, 1 with lithium carbonate and 1 with diazepam; 4 patients were not prescribed any psychotropic drug treatment. Five patients were prescribed a beta-blocking drug for hypertension. Of the 8 patients who experienced asystole, 3 were anaesthetised with propofol and 5 with etomidate. None of these 8 patients were on anti-epileptic medication and so they received the standard electrical stimulation protocol (rather than the higher doses administered to patients on anti-epileptics).

Table 2 shows that the time to first R-wave ranged from 0.04 to 9.12 seconds. Because of the nature of the titration procedure, most patients (*n* = 47) did not have a convulsion after the first stimulation and so experienced more than one electrical stimulation.

The data were analysed with each stimulus measured as an independent variable and the total number of stimulations given in the study was 172. Most patients (*n* = 49, 98%) experienced cerebral seizure activity (CSA) in this study and the one patient who did not underwent 5 electrical stimulations with no seizure. This patient did not experience asystole. The mean number of stimulations before experiencing CSA was 2.5. Our primary analysis was with asystole defined as 5 s without a QRS complex. With this definition, asystole occurred in 4/124 (3%) subconvulsive stimulations (95% CI 0.89 to 8.05%) and 5/48 (10%) convulsive stimulations (95% CI 3.47 to 22.66%). The number of subconvulsive stimulations is considerably higher than that of convulsive stimulations because, from the second stimulation onwards, the patient has experienced previous subconvulsive stimulations, but for convulsive stimulations the patient has not experienced any previous convulsive stimulations. Of the asystolic events, 6 occurred after a second electrical stimulation and the other 3 occurred after a third stimulation. Only one patient, a 70-year-old female with severe depression, experienced multiple episodes (2) of asystole, which were after her second (non-convulsive) and third (convulsive) electrical stimulation. The average age of the 8 patients who experienced asystole was 69.9 years and only 1 of them was male. Six of these patients were prescribed a combination of psychotropic medications and one was not prescribed any psychotropic drugs. Six of these patients had no documented history of cardiovascular disease.

Secondary analysis carried out using a definition of asystole of 7 s without a QRS complex reduced the number of episodes of asystole to 2. One of these followed a convulsive stimulation in a 72-year-old female with severe depression and a history of hypertension and ischaemic heart disease with previous stent insertion. She was the only patient (of three) taking a beta-blocker (atenolol) to exhibit asystole. The other episode of asystole lasting longer than 7 s was in a 70-year-old female with severe depression without a history of cardiovascular disease. It followed a second non-convulsive stimulation, lasted 9.12 s and was therefore the longest period of asystole noted.

A two-tailed Fisher’s exact test, comparing convulsive and subconvulsive stimulations, did not reach statistical significance (*P* = 0.12), but the odds ratio was 3.46 (95% CI 0.71 to 18.27) suggesting that a patient was more likely to have an episode of asystole after convulsive rather than subconvulsive stimulation. We compared the first and second stimulations in patients who had two or more subconvulsive stimulations, using a paired *t*-test, and found that the mean time to the first QRS complex was statistically longer after a second subconvulsive stimulus (*P* = 0.04, 95% CI –1.72 to –0.04). We then compared the first and second stimulations in all patients who had two or more stimulations, regardless of whether they experienced

**Table 1:** Demographic of the study sample

	Asystole	No asystole	Whole sample
Total *n*	8	42	50
			
Demographics			
, , , Male, *n* (%)	1 (12.5)	12 (28.6)	13 (26.0)
, , , Female, *n* (%)	7 (87.5)	30 (71.4)	37 (74.0)
, , , Age, mean (s.d. range): years	69.9 (54.0–85.7)	55.1 (38.8–71.3)	57.4 (40.5–74.3)
			
*Diagnosis*			
Depression, *n* (%)			
, , , Unspecified	2 (25.0)	4 (9.5)	6 (12.0)
, , , Mild	1 (12.5)	0	1 (2.0)
, , , Moderate	0	6 (14.3)	6 (12.0)
, , , Severe without psychosis	1 (12.5)	11 (26.2)	12 (24.0)
, , , Severe with psychosis	4 (50.0)	10 (23.8)	14 (28.0)
Bipolar affective disorder, *n* (%)			
, , , Moderate	0	1 (2.4)	1 (2.0)
, , , Severe without psychosis	0	4 (9.5)	4 (8.0)
, , , Severe with psychosis	0	2 (4.8)	2 (4.0)
Schizophrenia	0	2 (4.8)	2 (4.0)
Schizophrenia	0	2 (4.8)	2 (4.0)
Schizophrenia + severe depression	0	1 (2.4)	1 (2.0)
Severe depression with psychosis + Alzheimer’s disease	0	1 (2.4)	1 (2.0)
			
Psychotropic drugs, *n* (%)			
, , , No	1 (12.5)	3 (7.1)	4 (8.0)
, , , Single	1 (12.5)	7 (16.7)	8 (16.0)
, , , Multiple	6 (75.0)	32 (76.2)	38 (76.0)
			
History of cardiovascular diseaes, *n* (%)	2 (25.0)	10 (23.8)^a^	12 (24.0)
			
Beta-blocker prescribed, *n* (%)	1 (12.5)	4 (9.5)	5 (10.0)
			
Anaesthetic agent, *n* (%)			
, , , Propofol	3 (37.5)	9 (21.4)	12 (24.0)
, , , Etomidate	5 (62.5)	33 (78.6)	38 (76.0)
			
Type of ECT, *n* (%)			
, , , Bilateral	8 (100.0)	39 (92.9)	47 (94.0)
, , , Right unilateral	0	3 (7.1)	3 (6.0)

ECT, electroconvulsive therapy.

a. Including pulmonary embolism + one decision based on medications suggestive of cardiovascular disease.

**Table 2:** Asystole in convulsive and subconvulsive stimuli

	Cerebral seizure activity induced				No cerebral seizure activity induced			
Stimulation	Convulsive stimulations *n*	Time to first QRS, median (range)^a^	Asystole *n*	Proportion (95% CI)	Subconvulsive stimulations *n*	Time to first QRS, median (range)^a^	Asystole *n*	Proportion (95% CI)
1st	3	2.36 (0.88–3.00)	0	0.00 (0–0.71)	46 (+1 off-page)^b^	1.32 (0.04–4.84)	0	0.00 (0–0.08)
								
2nd	20	1.18 (0.04–7.08)	2	0.10 (0.01–0.32)	27	1.48 (0.04–9.12)	4	0.15 (0.04–0.34)
								
3rd	21 (+1 unreadable)^c^	1.88 (0.04–6.96)	3	0.14 (0.03–0.36)	5	1.36 (0.56–3.84)	0	0.00 (0–0.52)
								
4th	4	1.52 (0.04–4.44)	0	0.00 (0–0.6)	1	0.56	0	0.01 (0–0.98)
								
5th	0	0	0	0.00	1	0.96	0	0.01 (0–0.98)
								
**Total**	**48**			**0.10** **(0.03-0.23)**	**124**			**0.03** **(0.01-0.08)**

a. Time given in seconds.

b. Off-page: an ECG trace that went off the side of the page and could not be analysed.

c. Unreadable: an ECG trace so distorted it could not be analysed.

seizure activity (again using a paired *t*-test), and again found that the mean time to first QRS post-stimulation was significantly longer after a second stimulation (*P* = 0.03, 95% CI –1.09 to –0.05). We also compared the time to the first QRS complex after second and third stimulations in all patients who underwent three or more stimulations, using a paired *t*-test, but did not find a statistically significant difference (*P* = 0.36, 95% CI –0.66 to 0.96).

All episodes of asystole in this study resolved spontaneously without medical intervention.

## Discussion

Asystole (5 s without a QRS complex) occurred in 9 of 172 stimulations in this study (5%; 95% CI 0.02 to 0.10). As each episode of asystole resolved without medical intervention, our findings suggest that the empirical measurement of seizure threshold does not add to the cardiovascular risk of ECT, nor is there a need to routinely administer an anticholinergic drug. Contrary to expectation, asystole was more prevalent after convulsive than non-convulsive stimulation. We have also shown that time to the first QRS complex post-stimulation was longer in patients who received two subconvulsive stimulations rather than one. As increasing doses of electricity are given on subsequent stimulations when titrating up to seizure threshold, this could suggest that time to the first QRS complex simply increases together with the dose of electricity. This is supported by the fact that none of the episodes of asystole occurred after a patient’s first electrical stimulation and that the comparison of first and second stimulations, regardless of whether or not seizure activity ensued, showed a statistically significant difference between the times to first QRS complex (*P* = 0.03, 95% CI –1.09 to –0.05). On the other hand, there was no statistically significant difference between second and third stimulations (*P* = 0.36, 95% CI –0.66 to 0.96). This may be because the effect of increasing doses of electricity and/or absence of seizure activity is lost after a certain threshold, or because neither the electrical dose nor presence or absence of seizure activity are factors influencing the risk of asystole and the effect seen at previous stimulations may be due to small sample size. Further studies with a larger sample size would help to delineate this.

Our results support those of Burd & Kettl,^7^ who found that although asystole was common in elderly patients undergoing ECT (364/1146, 40.1%), routine use of atropine was unnecessary because brief asystole was not associated with adverse outcome. Burd & Kettl studied patients throughout ECT treatment courses, not just during stimulus titration, which may explain the difference in incidence of asystole observed in our study. They also refer to reports of asystole lasting up to 7 s and so we attempted to conduct a secondary analysis using a definition of 7 s without a QRS complex. However, in our sample there were only 2 episodes of asystole which exceeded 7 s (1 in a convulsive stimulation and 1 in which no convulsion was stimulated) and so no statistical analysis of these episodes could be performed. In 1996, McCall *et al*^10^ used an even more conservative definition of asystole (10 s of ECG electrical silence) in an attempt to capture only pathological asystolic events. Using this cut-off there were no patients in our study who experienced asystole, again supporting the idea that the periods of electrical silence in our study were not pathological. Furthermore, only one of the patients in our study suffered more than one episode of asystole, which suggests that, for a given individual, one episode of asystole does not generally predict further similar events. A limitation of our study was that it was not possible to statistically analyse the other factors that could prolong the time to the first QRS complex, because of the small number of patients. Larger prevalence studies will be needed to further investigate these factors.
